# Personal

**Published:** 1905-04

**Authors:** 


### PERSONAL.

Dr. G. E. Beebe, late of the staff of the New York Manhattan Eye and Ear Hospital, has returned to Buffalo, and is located at No. 120 North Pearl street, above Allen. Her practice is limited to diseases of the eye and refraction. Hours: 9 a. m. to 1 p. m., and by appointment. Frontier telephone, 3145.

---

Dr. Albert Vander Veer, of Albany, has been appointed by Governor Higgins a delegate to represent the state of New York, at a meeting of the American Medical Association council on medical education to be held at Chicago, April 20, 1905. Dr. Vander Veer has been a leader in medical education affairs for more than twenty-five years, during which time he has been a champion of everything relating to improvement and progress in this important question.

---

Dr. Julius Ullman, of Buffalo, has been appointed a special examiner of applicants for admission to the state hospital for consumptives at Raybrook. Dr. John H. Pryor, formerly of Buffalo, is the superintendent of the state hospital. Dr. Ullman's appointment was made by the faculty of the University of Buffalo.

---

Dr. A. R. Cushny, of the University of Michigan, Ann Arbor, has been appointed professor of pharmacology and materia medica in University College, London.

---

Dr. H. C. Buswell, of Buffalo, who has been traveling in Europe for a year, has returned to his home and resumed his medical practice.

---

Dr. Charles W. Banta, who went to Europe with Dr. Buswell, has remained at Berne to pursue the further study of surgery under the tutelage of Professor Theodor Kocher.

Dr. Magnus A. Tate, of Cincinnati, was elected president of the Cincinnati Academy of Medicine at its recent annual meeting. Dr. Tate was installed into office March 13, 1905. At this meeting the vote of the academy was taken as to whether in the future the Ohio State Society, of which the academy is a part, should publish its transactions in book form, as heretofore, or start an official journal. By an overwhelming majority the academy voted in favor of publishing the transactions in annual book form, thus continuing the present method.

---

Dr. Joseph D. Bryant, of New York, president of the State Medical Society, has gone to Florida in company with former President Grover Cleveland on a three weeks' hunting and fishing tour, on which they started March 20, 1905.

---

Dr. Jacob Miller, of Buffalo, was appointed by the Fire Commissioners temporary surgeon to the department to fill the vacancy created by the death of Dr. E. C. W. O'Brien. The permanent appointment will be made from the eligible civil service list when certified.

